# Closed-loop approach to tuning deep brain stimulation parameters for Parkinson's disease

**DOI:** 10.1186/1471-2202-16-S1-F2

**Published:** 2015-12-18

**Authors:** Abbey B Holt, Max Shinn, Theoden I Netoff

**Affiliations:** 1 Graduate Program in Neuroscience, University of Minnesota, Minneapolis, MN, 55455, USA; 2 Department of Neuroscience, University of Minnesota, Minneapolis, MN, 55455, USA; 3 Department of Biomedical Engineering, University of Minnesota, Minneapolis, MN, 55455, USA

## 

Deep brain stimulation (DBS) is used to treat motor symptoms of patients with Parkinson's disease (PD). However, tuning stimulation parameters is currently done using a time intensive trial-and-error process until maximum therapy is achieved with minimal side effects [1]. There is a need for a systematic approach to tuning parameters based on patient physiology. With the development of DBS electrodes that can simultaneously stimulate and record [2], a closed-loop approach may be taken. It is hypothesized that emergent oscillations in the basal ganglia network, particularly in the beta range (12-35 Hz) lead to motor symptoms of PD [3], and that DBS works by disrupting these oscillations. Our hypothesis is that stimulating at a specific phase in the pathological oscillation will optimally disrupt the oscillatory activity, and that this phase can be predicted from the phase response curve (PRC). Here, we use a computational network model of PD with an emergent pathological 34 Hz oscillation [4] to test this closed-loop approach to DBS and confirm the results in vitro. By stimulating at a specific phase in the beta oscillation we are able to modulate the power of the oscillation in the model. By stimulating soon after the peak in the oscillation, we disrupt the 34 Hz oscillation, while stimulating later in the period enhances it. Hence, the timing of stimulation affects how well the population of neurons desynchronized. Next, we test this concept in vitro by synchronizing patch-clamped neurons in the substantia nigra pars reticulata (an output nucleus of the basal ganglia) to an oscillatory input, such as a beta oscillation. We show that stimulating at a particular phase of the oscillatory input affects how well neurons synchronize or desynchronize to that input. Finally, we show it is possible to use the PRC to predict how stimulating at a specific phase will affect the neuron's ability to synchronize or desynchronize from the oscillatory input in vitro.

This work shows that stimulating at specific phases in an oscillation can synchronize or desynchronize neurons in a computational model and in vitro. By stimulating at specific phases of an emergent pathological oscillation in a closed-loop approach to DBS, we were able to suppress a pathological oscillation in a computational model of PD. In this approach, a frequency of 34 Hz was used for DBS, which is much lower than the value used clinically (>100 Hz). Through closed-loop stimulation, precisely timed stimuli with respect to the phase of the oscillation can dramatically decrease stimulus power needed for DBS. The ability to synchronize or desynchronize a neuron to an oscillatory input by stimulating at a certain phase was also validated in vitro. It is possible to predict the phase of stimulation to maximally disrupt neuronal synchronization to an external oscillatory input in single neurons using a PRC. We have previously shown a novel method to estimate a PRC from population data [5] in a computational model of PD. This suggests it may be possible to predict the phase at which to stimulate in order to optimally disrupt a pathological population oscillation in PD using the PRC, and apply this in a closed-loop approach to DBS.
